# Methyl 2-meth­oxy-4-{[2-(4-nitro­phen­yl)hydrazinyl­idene]meth­yl}benzoate

**DOI:** 10.1107/S160053681003802X

**Published:** 2010-09-30

**Authors:** Zhen-xin Zhao, He-ping Li, Bu-wei Ma

**Affiliations:** a Department of Chemistry and Chemical Engineering, Henan University of Urban Construction, Pingdingshan 467044, People’s Republic of China; b School of Chemistry and Biological Engineering, Guilin University of Technology, People’s Republic of China; c Department of Architectural Environment, and Energy Engineering, Henan University of Urban Construction, Pingdingshan 467044, People’s Republic of China

## Abstract

The mol­ecule of the title Schiff base compound, C_16_H_15_N_3_O_5_, obtained from a condensation reaction of 4-acet­oxy-3-meth­oxy­benzaldehyde and 4-nitro­phenyl­hydrazine, adopts an *E* geometry with respect to the C=N double bond. The mol­ecule is roughly planar, with the two benzene rings twisted slightly with respect to each other by a dihedral angle of 6.90 (9)°. In the crystal, inter­molecular N—H⋯O hydrogen bonds link centrosymmetrically related pairs of mol­ecules, forming dimers of *R*_2_^2^(22) graph-set motif. The dimers are further connected through slipped π–π inter­actions between symmetry-related benzene rings [centroid–centroid distance of 3.646 (1) Å, offset angle of 15.4°].

## Related literature

For potential applications of hydrazone derivatives, see: Okabe *et al.* (1993 ▶). For related structures, see: Szczesna & Urbanczyk-Lipkowska (2002 ▶); Zhen & Han (2005 ▶); Kuleshova *et al.* (2003 ▶); Baughman *et al.* (2004 ▶). For hydrogen-bond motifs, see: Etter *et al.* (1990 ▶); Bernstein *et al.* (1994 ▶).
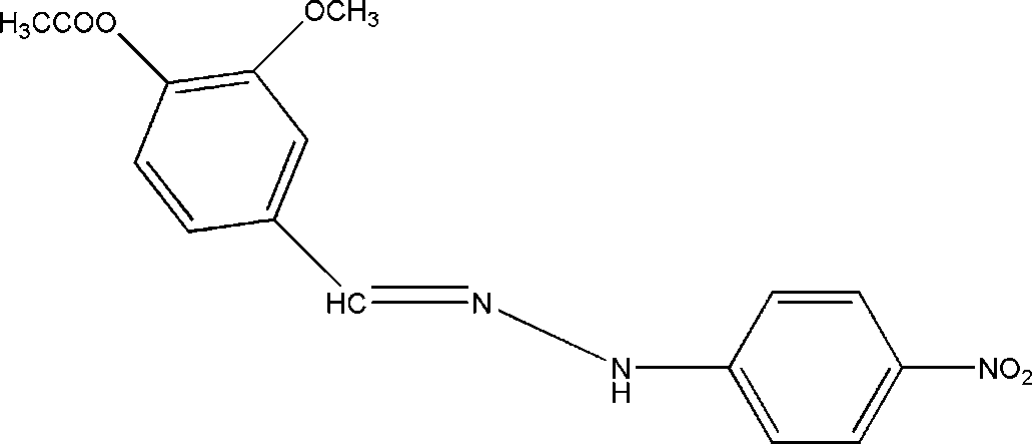


## Experimental

### 

#### Crystal data

C_16_H_15_N_3_O_5_
*M* *_r_* = 329.31
Monoclinic, 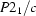

*a* = 8.5983 (7) Å
*b* = 14.6982 (9) Å
*c* = 13.2096 (10) Å
β = 107.860 (9)°
*V* = 1589.0 (2) Å^3^
*Z* = 4
Mo *K*α radiation
μ = 0.10 mm^−1^
*T* = 293 K
0.23 × 0.20 × 0.19 mm

#### Data collection

Bruker SMART CCD area-detector diffractometer
Absorption correction: multi-scan (*SADABS*; Bruker, 1998 ▶) *T*_min_ = 0.971, *T*_max_ = 0.976
6773 measured reflections
3255 independent reflections
1397 reflections with *I* > 2σ(*I*)
*R*_int_ = 0.029

#### Refinement

*R*[*F* ^2^ > 2σ(*F* ^2^)] = 0.036
*wR*(*F* ^2^) = 0.072
*S* = 0.72
3255 reflections
219 parameters
H-atom parameters constrained
Δρ_max_ = 0.13 e Å^−3^
Δρ_min_ = −0.16 e Å^−3^

### 

Data collection: *SMART* (Bruker, 1998 ▶); cell refinement: *SAINT* (Bruker, 1998 ▶); data reduction: *SAINT*; program(s) used to solve structure: *SHELXTL* (Sheldrick, 2008 ▶); program(s) used to refine structure: *SHELXL97* (Sheldrick, 2008 ▶); molecular graphics: *ORTEPIII* (Burnett & Johnson, 1996 ▶), *ORTEP-3 for Windows* (Farrugia, 1997 ▶) and *PLATON* (Spek, 2009 ▶); software used to prepare material for publication: *SHELXTL*.

## Supplementary Material

Crystal structure: contains datablocks global, I. DOI: 10.1107/S160053681003802X/dn2604sup1.cif

Structure factors: contains datablocks I. DOI: 10.1107/S160053681003802X/dn2604Isup2.hkl

Additional supplementary materials:  crystallographic information; 3D view; checkCIF report

## Figures and Tables

**Table 1:** Hydrogen-bond geometry (Å, °)

*D*—H⋯*A*	*D*—H	H⋯*A*	*D*⋯*A*	*D*—H⋯*A*
N1—H1⋯O5^i^	0.86	2.29	3.0068 (19)	141

Symmetry code: (i) 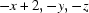.

**Fig. 1.**
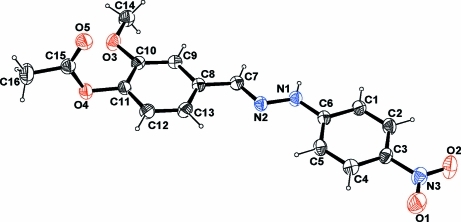
Molecular view of the title compound with the atom labeling scheme. Displacement ellipsoids are drawn at the 30% probability level. H atoms are represented as small spheres of arbitrary radii.

**Fig. 2.**
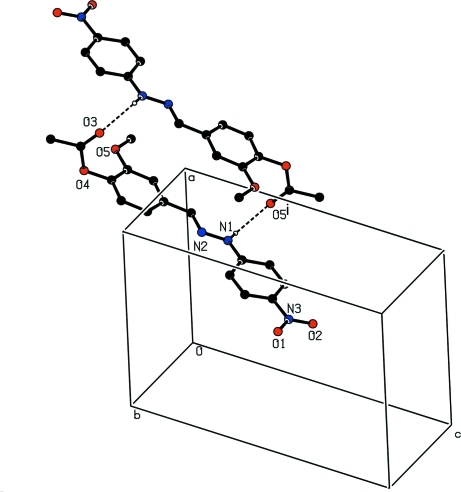
Partial packing view showing the formation of dimer through N-H···O hydrogen bonds shown as dashed lines. H atoms not involved in hydrogen bonding have been omitted for clarity. [Symmetry code: (i) -x+2, -y, -z]

## supplementary crystallographic information

### Comment

As some phenylhydrazone derivatives show potential application in biochemistry(Okabe *et al.*,1993), phenylhydrazone has recently attracted our attention. In this paper, we report the synthesis and crystal structure of the title compound.

The molecule adopts an E geometry with respect to the C=N double bond (Fig. 1). The methoxybenzene and the nitrobenzene rings are roughly planar, with however the two benzene rings slightly twisted with respect to each other by a dihedral angle of 6.90 (9)°. The geometry within the hydrazone moiety agrees with related compound found in the literature (Szczesna & Urbanczyk-Lipkowska, 2002; Zhen & Han, 2005; Kuleshova *et al.*, 2003; Baughman *et al.*, 2004.

Intermolecular N—H···O hydrogen bonds link the molecule two by two to form dimer (Table 1, Fig. 2) with R^2^_2_(22) graph set motif (Etter *et al.*, 1990); Bernstein *et al.*, 1994). The dimers are connected through slippest π-π interactions involving the symmetry related (1-x, -y, -z) C1-C6 and C8-C13 benzene rings with a centroid to centroid distance of 3.646 (1)Å and an interplanar distance of 3.515Å resulting in an offset angle of 15.4°.

### Experimental

4-Nitrophenylhydrazine (1 mmol, 0.153 g) was dissolved in anhydrous ethanol (10 ml), The mixture was stirred for several minitutes at 351k, 4-acetoxy-3-methoxybenzaldehyde(1 mmol, 0.194 g) in ethanol (8 mm l) was added dropwise and the mixture was stirred at refluxing temperature for 2 h. The product was isolated and recrystallized from methanol/dicholomethane(1:1), red single crystals of (I) was obtained after 3 d.

### Refinement

All H atoms attached to C atoms and N atom were fixed geometrically and treated as riding with C—H = 0.96 Å (methyl) or 0.93 Å (aromatic) and N—H = 0.86 Å with U_iso_(H) = 1.2U_eq_(C or N) or U_iso_(H) = 1.5U_eq_(C_methyl_).

### Crystal data

**Table:**

C_16_H_15_N_3_O_5_	*F*(000) = 688
*M**_r_* = 329.31	*D*_x_ = 1.377 Mg m^−^^3^
Monoclinic, *P*2_1_/*c*	Mo *K*α radiation, λ = 0.71073 Å
Hall symbol: -P 2ybc	Cell parameters from 1694 reflections
*a* = 8.5983 (7) Å	θ = 3.2–26.3°
*b* = 14.6982 (9) Å	µ = 0.10 mm^−^^1^
*c* = 13.2096 (10) Å	*T* = 293 K
β = 107.860 (9)°	Block, red
*V* = 1589.0 (2) Å^3^	0.23 × 0.20 × 0.19 mm
*Z* = 4	

### Data collection

**Table:**

Bruker SMART CCD area-detector diffractometer	3255 independent reflections
Radiation source: fine-focus sealed tube	1397 reflections with *I* > 2σ(*I*)
graphite	*R*_int_ = 0.029
ω scans	θ_max_ = 26.4°, θ_min_ = 3.2°
Absorption correction: multi-scan (*SADABS*; Bruker, 1998)	*h* = −10→10
*T*_min_ = 0.971, *T*_max_ = 0.976	*k* = −15→18
6773 measured reflections	*l* = −16→10

### Refinement

**Table:**

Refinement on *F*^2^	Primary atom site location: structure-invariant direct methods
Least-squares matrix: full	Secondary atom site location: difference Fourier map
*R*[*F*^2^ > 2σ(*F*^2^)] = 0.036	Hydrogen site location: inferred from neighbouring sites
*wR*(*F*^2^) = 0.072	H-atom parameters constrained
*S* = 0.72	*w* = 1/[σ^2^(*F*_o_^2^) + (0.032*P*)^2^] where *P* = (*F*_o_^2^ + 2*F*_c_^2^)/3
3255 reflections	(Δ/σ)_max_ = 0.007
219 parameters	Δρ_max_ = 0.13 e Å^−^^3^
0 restraints	Δρ_min_ = −0.16 e Å^−^^3^

### Special details

**Table:**

Geometry. All esds (except the esd in the dihedral angle between two l.s. planes) are estimated using the full covariance matrix. The cell esds are taken into account individually in the estimation of esds in distances, angles and torsion angles; correlations between esds in cell parameters are only used when they are defined by crystal symmetry. An approximate (isotropic) treatment of cell esds is used for estimating esds involving l.s. planes.
Refinement. Refinement of *F*^2^ against ALL reflections. The weighted *R*-factor *wR* and goodness of fit *S* are based on *F*^2^, conventional *R*-factors *R* are based on *F*, with *F* set to zero for negative *F*^2^. The threshold expression of *F*^2^ > σ(*F*^2^) is used only for calculating *R*-factors(gt) *etc*. and is not relevant to the choice of reflections for refinement. *R*-factors based on *F*^2^ are statistically about twice as large as those based on *F*, and *R*- factors based on ALL data will be even larger.

### Fractional atomic coordinates and isotropic or equivalent isotropic displacement parameters (Å^2^)

**Table:**

	*x*	*y*	*z*	*U*_iso_*/*U*_eq_	
O1	0.3583 (2)	0.28271 (10)	0.40773 (12)	0.0934 (5)	
O2	0.41338 (19)	0.17034 (10)	0.51701 (12)	0.0912 (5)	
O3	0.94760 (16)	−0.07061 (8)	−0.28742 (9)	0.0600 (4)	
O4	0.84502 (16)	0.09303 (8)	−0.37261 (9)	0.0560 (4)	
O5	1.10849 (17)	0.12748 (8)	−0.29655 (11)	0.0628 (4)	
N1	0.66329 (16)	0.00919 (9)	0.15774 (11)	0.0493 (4)	
H1	0.6925	−0.0461	0.1750	0.059*	
N2	0.68044 (17)	0.04452 (9)	0.06596 (11)	0.0451 (4)	
N3	0.4121 (2)	0.20643 (13)	0.43252 (14)	0.0649 (5)	
C1	0.6057 (2)	0.02353 (12)	0.32125 (14)	0.0522 (5)	
H1B	0.6504	−0.0339	0.3408	0.063*	
C2	0.5452 (2)	0.07193 (13)	0.38904 (14)	0.0553 (5)	
H2B	0.5495	0.0478	0.4549	0.066*	
C3	0.4777 (2)	0.15662 (12)	0.35965 (15)	0.0468 (5)	
C4	0.4691 (2)	0.19305 (11)	0.26147 (14)	0.0495 (5)	
H4A	0.4215	0.2498	0.2416	0.059*	
C5	0.5311 (2)	0.14503 (11)	0.19376 (14)	0.0468 (5)	
H5A	0.5266	0.1696	0.1280	0.056*	
C6	0.6006 (2)	0.06010 (12)	0.22253 (14)	0.0417 (4)	
C7	0.7483 (2)	−0.00733 (11)	0.01437 (13)	0.0452 (5)	
H7A	0.7806	−0.0654	0.0403	0.054*	
C8	0.7767 (2)	0.02182 (11)	−0.08375 (13)	0.0403 (4)	
C9	0.85046 (19)	−0.03850 (11)	−0.13560 (13)	0.0434 (5)	
H9A	0.8822	−0.0956	−0.1061	0.052*	
C10	0.8777 (2)	−0.01552 (11)	−0.23029 (14)	0.0434 (5)	
C11	0.8294 (2)	0.06963 (12)	−0.27239 (13)	0.0449 (5)	
C12	0.7565 (2)	0.13013 (11)	−0.22286 (15)	0.0518 (5)	
H12A	0.7246	0.1870	−0.2528	0.062*	
C13	0.7300 (2)	0.10665 (11)	−0.12796 (15)	0.0516 (5)	
H13A	0.6809	0.1479	−0.0939	0.062*	
C14	0.9936 (2)	−0.15954 (11)	−0.24715 (14)	0.0632 (6)	
H14A	1.0419	−0.1913	−0.2935	0.095*	
H14B	0.8987	−0.1920	−0.2433	0.095*	
H14C	1.0714	−0.1554	−0.1774	0.095*	
C15	0.9935 (3)	0.12176 (12)	−0.37443 (17)	0.0507 (5)	
C16	0.9907 (3)	0.14511 (15)	−0.48491 (15)	0.0823 (7)	
H16A	0.9030	0.1868	−0.5155	0.123*	
H16B	0.9748	0.0907	−0.5271	0.123*	
H16C	1.0927	0.1728	−0.4832	0.123*	

### Atomic displacement parameters (Å^2^)

**Table:**

	*U*^11^	*U*^22^	*U*^33^	*U*^12^	*U*^13^	*U*^23^
O1	0.1226 (15)	0.0764 (11)	0.0863 (11)	0.0205 (11)	0.0397 (10)	−0.0154 (10)
O2	0.1189 (14)	0.1058 (12)	0.0648 (10)	−0.0012 (10)	0.0517 (10)	−0.0052 (9)
O3	0.0790 (10)	0.0505 (7)	0.0600 (9)	0.0083 (7)	0.0352 (8)	0.0004 (7)
O4	0.0524 (9)	0.0728 (9)	0.0439 (8)	−0.0030 (8)	0.0165 (7)	0.0089 (7)
O5	0.0551 (9)	0.0710 (9)	0.0609 (9)	−0.0025 (8)	0.0159 (8)	0.0054 (8)
N1	0.0584 (11)	0.0466 (9)	0.0481 (10)	0.0032 (8)	0.0239 (9)	0.0052 (8)
N2	0.0454 (10)	0.0511 (9)	0.0398 (9)	−0.0051 (8)	0.0144 (8)	0.0022 (8)
N3	0.0633 (13)	0.0740 (13)	0.0596 (13)	−0.0106 (11)	0.0220 (11)	−0.0172 (11)
C1	0.0561 (14)	0.0545 (11)	0.0498 (12)	0.0049 (10)	0.0219 (11)	0.0108 (10)
C2	0.0574 (14)	0.0680 (13)	0.0445 (12)	−0.0063 (11)	0.0214 (11)	0.0074 (11)
C3	0.0452 (13)	0.0533 (12)	0.0450 (12)	−0.0092 (10)	0.0186 (10)	−0.0085 (10)
C4	0.0509 (14)	0.0436 (11)	0.0530 (12)	−0.0085 (10)	0.0145 (11)	−0.0036 (10)
C5	0.0546 (13)	0.0444 (11)	0.0428 (11)	−0.0092 (10)	0.0172 (10)	0.0019 (9)
C6	0.0398 (11)	0.0472 (11)	0.0379 (11)	−0.0100 (9)	0.0117 (9)	−0.0017 (9)
C7	0.0427 (13)	0.0459 (11)	0.0464 (12)	−0.0054 (10)	0.0131 (10)	−0.0006 (9)
C8	0.0397 (12)	0.0405 (10)	0.0408 (11)	−0.0068 (9)	0.0124 (10)	−0.0009 (9)
C9	0.0434 (13)	0.0392 (10)	0.0472 (12)	−0.0011 (9)	0.0131 (10)	−0.0007 (9)
C10	0.0409 (12)	0.0441 (11)	0.0458 (12)	−0.0023 (10)	0.0144 (10)	−0.0066 (10)
C11	0.0417 (12)	0.0540 (12)	0.0386 (11)	−0.0017 (10)	0.0117 (10)	0.0044 (10)
C12	0.0548 (13)	0.0447 (11)	0.0599 (13)	0.0036 (10)	0.0238 (11)	0.0082 (10)
C13	0.0561 (13)	0.0457 (11)	0.0592 (13)	0.0017 (10)	0.0268 (11)	−0.0035 (10)
C14	0.0789 (16)	0.0483 (11)	0.0683 (14)	0.0097 (11)	0.0316 (13)	−0.0045 (11)
C15	0.0583 (14)	0.0454 (11)	0.0525 (13)	0.0064 (11)	0.0232 (12)	0.0019 (10)
C16	0.0886 (18)	0.1103 (17)	0.0573 (14)	−0.0082 (15)	0.0361 (13)	0.0100 (13)

### Geometric parameters (Å, °)

**Table:**

O1—N3	1.2188 (18)	C5—C6	1.386 (2)
O2—N3	1.2326 (18)	C5—H5A	0.9300
O3—C10	1.3655 (18)	C7—C8	1.455 (2)
O3—C14	1.4210 (18)	C7—H7A	0.9300
O4—C15	1.352 (2)	C8—C13	1.383 (2)
O4—C11	1.4135 (18)	C8—C9	1.387 (2)
O5—C15	1.191 (2)	C9—C10	1.383 (2)
N1—C6	1.3663 (18)	C9—H9A	0.9300
N1—N2	1.3678 (16)	C10—C11	1.381 (2)
N1—H1	0.8600	C11—C12	1.365 (2)
N2—C7	1.2766 (18)	C12—C13	1.385 (2)
N3—C3	1.454 (2)	C12—H12A	0.9300
C1—C2	1.366 (2)	C13—H13A	0.9300
C1—C6	1.399 (2)	C14—H14A	0.9600
C1—H1B	0.9300	C14—H14B	0.9600
C2—C3	1.378 (2)	C14—H14C	0.9600
C2—H2B	0.9300	C15—C16	1.492 (2)
C3—C4	1.384 (2)	C16—H16A	0.9600
C4—C5	1.369 (2)	C16—H16B	0.9600
C4—H4A	0.9300	C16—H16C	0.9600
			
C10—O3—C14	117.25 (13)	C9—C8—C7	118.60 (16)
C15—O4—C11	117.01 (14)	C10—C9—C8	121.35 (16)
C6—N1—N2	121.19 (14)	C10—C9—H9A	119.3
C6—N1—H1	119.4	C8—C9—H9A	119.3
N2—N1—H1	119.4	O3—C10—C11	116.35 (16)
C7—N2—N1	115.96 (14)	O3—C10—C9	125.47 (16)
O1—N3—O2	122.44 (18)	C11—C10—C9	118.18 (16)
O1—N3—C3	118.54 (18)	C12—C11—C10	121.53 (17)
O2—N3—C3	119.02 (19)	C12—C11—O4	118.68 (16)
C2—C1—C6	120.11 (17)	C10—C11—O4	119.64 (16)
C2—C1—H1B	119.9	C11—C12—C13	119.91 (16)
C6—C1—H1B	119.9	C11—C12—H12A	120.0
C1—C2—C3	119.87 (17)	C13—C12—H12A	120.0
C1—C2—H2B	120.1	C8—C13—C12	120.00 (16)
C3—C2—H2B	120.1	C8—C13—H13A	120.0
C2—C3—C4	120.70 (17)	C12—C13—H13A	120.0
C2—C3—N3	118.93 (18)	O3—C14—H14A	109.5
C4—C3—N3	120.36 (18)	O3—C14—H14B	109.5
C5—C4—C3	119.54 (17)	H14A—C14—H14B	109.5
C5—C4—H4A	120.2	O3—C14—H14C	109.5
C3—C4—H4A	120.2	H14A—C14—H14C	109.5
C4—C5—C6	120.43 (16)	H14B—C14—H14C	109.5
C4—C5—H5A	119.8	O5—C15—O4	123.01 (18)
C6—C5—H5A	119.8	O5—C15—C16	126.1 (2)
N1—C6—C5	122.77 (16)	O4—C15—C16	110.93 (19)
N1—C6—C1	117.89 (16)	C15—C16—H16A	109.5
C5—C6—C1	119.33 (17)	C15—C16—H16B	109.5
N2—C7—C8	121.93 (16)	H16A—C16—H16B	109.5
N2—C7—H7A	119.0	C15—C16—H16C	109.5
C8—C7—H7A	119.0	H16A—C16—H16C	109.5
C13—C8—C9	119.02 (16)	H16B—C16—H16C	109.5
C13—C8—C7	122.37 (16)		

### Hydrogen-bond geometry (Å, °)

**Table:**

*D*—H···*A*	*D*—H	H···*A*	*D*···*A*	*D*—H···*A*
N1—H1···O5^i^	0.86	2.29	3.0068 (19)	141

Symmetry codes: (i) −*x*+2, −*y*, −*z*.
